# Information-Theoretical Analysis of the Cycle of Creation of Knowledge and Meaning in Brains under Multiple Cognitive Modalities

**DOI:** 10.3390/s24051605

**Published:** 2024-02-29

**Authors:** Joshua J. J. Davis, Florian Schübeler, Robert Kozma

**Affiliations:** 1 Dodd-Walls Centre for Photonics and Quantum Technologies, Department of Physics & Ian Kirk’s Lab., Centre for Brain Research, The University of Auckland, Auckland 1142, New Zealand; joshua.davis@auckland.ac.nz; 2 The Embassy of Peace, Whitianga, Coromandel 3591, New Zealand; flosued@gmail.com; 3 Department of Mathematics, University of Memphis, Memphis, TN 38152, USA; 4 School of Informatics, Obuda University, H-1034 Budapest, Hungary; 5 Kozmos Research Laboratories, Boston, MA 02215, USA

**Keywords:** EEG, cognition, meaning, knowledge, intentional action, pragmatic information, meditation, awareness

## Abstract

It is of great interest to develop advanced sensory technologies allowing non-invasive monitoring of neural correlates of cognitive processing in people performing everyday tasks. A lot of progress has been reported in recent years in this research area using scalp EEG arrays, but the high level of noise in the electrode signals poses a lot of challenges. This study presents results of detailed statistical analysis of experimental data on the cycle of creation of knowledge and meaning in human brains under multiple cognitive modalities. We measure brain dynamics using a HydroCel Geodesic Sensor Net, 128-electrode dense-array electroencephalography (EEG). We compute a pragmatic information (PI) index derived from analytic amplitude and phase, by Hilbert transforming the EEG signals of 20 participants in six modalities, which combine various audiovisual stimuli, leading to different mental states, including relaxed and cognitively engaged conditions. We derive several relevant measures to classify different brain states based on the PI indices. We demonstrate significant differences between engaged brain states that require sensory information processing to create meaning and knowledge for intentional action, and relaxed-meditative brain states with less demand on psychophysiological resources. We also point out that different kinds of meanings may lead to different brain dynamics and behavioral responses.

## 1. Introduction

Intensive research has been conducted in recent years to develop advanced sensory technologies allowing non-invasive monitoring and classification of neural correlates of cognitive processing in human brains via EEG with the aid of convolutional neural networks [1], for example. Some interesting studies have focused on exploring neural correlates of learning and working memory tasks, for example, with the use of EEG to compare several classifiers [2]. As indicated by [3], EEG technologies have some advantages and good applications for Developmental Cognitive Neuroscience, while new trends in quantitative analysis are emerging in a broad spectrum of applications, including medical applications [4]. One important area of research is the study of brain dynamics in relation to motor tasks and rehabilitation [5].

Significant progress has been made using scalp EEG arrays for cognitive state identification of people performing everyday tasks, some of them in sport [6] and meditation [7], and for mental state detection for pilots and accident prevention [8]. Studies have also involved participants tested with closed vs. open eyes in visual and auditory recognition tasks, as well as motor tasks [9,10] including emotion recognition in human computer interaction [11]. Several challenges in different areas are reviewed in [12] that included safety and health, for example, via measuring physiological signals such as EEG. Other authors have explored the challenges associated with brain computer interface for emotion recognition [13,14] associated with machine learning and other models [15], as for example, in planning and rational decision making [16], something that requires robust classification methods for EEG signals in emotion recognition, as discussed by [17]. Another relevant field of study concerning various physiological signals concerns the characterization of mental stress and fatigue, as described in studies like [18] where electrodermal activity is measured to classify calm from distress conditions. This has also been discussed with the use of EEG in cooperative game theory and fatigue [19], as well as mental stress and its influence on neural coordination [20].

Intentional action and values-based decision making necessitate meaningful mental frameworks [21,22]. Therefore, in considering meaning and values, we must take into consideration the interaction of the brain and the mind in creating semantic, visual, aesthetic, scientific, musical, and spiritual meanings, for example. When we value someone or something, this person or thing becomes meaningful to us. It is of great importance to study the neural processes underlying the creation of meaning in the human cortex.

Intentional neurodynamics is a key component of the emergence of meaning in human brains, as described by Freeman [23]. An important aspect of intentionality is the unity of brain, mind, and body, which has been described in the early works of Thomas Aquinas [24]. The traditions of Aquinas were followed by Brentano [25], describing the creation of meaning in spiritual experiences; see also [26,27]. Considering the unity of brain, mind, and body, the energy consumption of the brain during the creation of meaning becomes an important research area [28,29]. Freeman’s pioneering experimental studies on spatio-temporal oscillations in brains produced several key findings, including the identification of sequences of metastable amplitude-modulated activity patterns (wave packets), which are associated with the meaning of the sensory experiences and produce consecutive meaningful decisions and actions [30,31,32]. The formation of the sequence of such brain activity patterns impacts the energy utilization of the brain, which in turn provides a potential tool to monitor cognitive processing.

Modern brain imaging techniques allow monitoring the role of various brain areas during cognitive processing. Depending on the nature of the cognitive activities, the measured patterns of brain oscillations would change. For example, it is expected to observe markedly different patterns during relaxed states, as compared with more active states, such as solving mathematical tasks, identifying visual clues. It is expected that more engaged cognitive states likely require more metabolic energy than relaxed ones. From our perspective, the cognitive activities using various sensory stimuli and leading to intentional actions are especially important, as they are related to the meaning of the stimuli in the context of the actual physical and mental state of the individual.

It is important to extend the results of previous studies, which used invasive electrocorticogram (ECoG) arrays in animal brains [33], to the domain of non-invasive scalp monitoring with humans. Successes of those efforts would produce crucial progress in human brain studies by providing powerful tools to quantify the creation of meaning and knowledge in human brains, using statistical and information-theoretical indices. The challenges are daunting, as the extraction of meaning from scalp EEG signals appears to be intractable due to, for example, the drastic deterioration of the brain electric signals after passing through the skull, amongst other challenges. Nonetheless, a breakthrough in this problem is possible, based on advanced signal processing and information-theoretical analysis [34,35,36].

In this paper, we introduce the action perception cycle, by which a stimulus becomes meaningful and is selected by the subject’s cerebral cortex, which creates the network structures and dynamics required for decision making, intentional action, and behavior [23,24]. We expand the preliminary analysis of EEG measurements reported in [37]. We employ the Hilbert transform to obtain the analytic amplitude (AA), analytic phase (AP), and instantaneous frequency (IF) of the highly nonlinear, nonstationary EEG signals, and derive pragmatic information (PI) indices, which were originally introduced for lasers [38,39], and extended to brain dynamics [33,34,40,41]. Next, we present EEG experiments based on human participants in different modalities, collected in Ian J. Kirk’s Lab, Centre for Brain Research at The University of Auckland in New Zealand [42,43]. Previously, we reported results of EEG measurements with two modalities, meditation and video watching [36], which are now extended to six modalities [37], including meditation, scrambled words, video watching, ambiguous images, math mind, and sentences, for a comprehensive and robust analysis. We apply mathematical and statistical analysis using pragmatic information indices to evaluate the EEG experimental data. The obtained results demonstrate that the behavior of such indices correlates with the formation of meaning in neural structures and energy consumption levels in the brain. Finally, we outline perspectives and directions for future research.

## 2. Materials and Methods

### 2.1. Data Acquisition

Experiments were conducted using Electrical Geodesics Inc (EGI, Eugene, OR, USA) 128-channel scalp EEG electrode arrays [44], following the basic methodology for experimental design and data acquisition described in [36] with 20 participants. Expanding on the previous results using only 2 modalities (meditation and video watching) with 20 participants, here we describe the outcomes corresponding to 6 modalities:

**Meditation (MED):** Participants were requested to conduct meditation of their choice for 7 min. In the case of participants with little or no meditation experience, they were asked to relax with their eyes closed for 7 min.

**Scrambled Words (WORDS):** Participants were presented twenty (20) scrambled words that pertained to one (1) of the two (2) categories, either a value, such as Love or Truth, for example, or an object from nature, such as Sand or Pebble. The participants were requested to try to identify the original word (unscramble it). Once they had established the category of the word, they were asked to press number 1 on the numerical keypad when they identified the word describing a “Value” or to press number 2 on the numerical keypad when the word was describing an “Object”.

**Figure 1:**
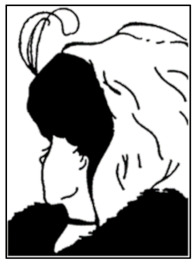
An example of the ambiguous images shown to the participants. (My Wife and My Mother-In-Law, W. E. Hill, 1915).

**Ambiguous Images (IMG):** We presented twelve (12) images with ambiguous content to the participants and asked them to specify how many individual images they could find by pressing a number between 1 and 9 on the numerical keypad to identify the number of images found; see Figure 1 with two (2) different images in it, for an example.

**Math Mind (MM):** For this modality, we recorded a pleasant female voice presenting twenty-eight (28) simple arithmetic operations that the participants were asked to resolve in their mind by applying some rules, until they would reach a solution with a single digit between one (1) and nine (9), for each arithmetic operation. For example, the participant would hear “7 times 7”. This equates to 49, a two-digit result. Now the participant would add these two digits together (i.e., 4 + 9 = 13) and repeat this step until they reach a single digit result, in this case the answer would be “4”, after the final computation of “1 + 3” was performed.

**Sentences (SENT):** Twenty (20) sentences were recorded with the same female voice as for the MM modality. The sentence was comprised of either a positive statement, such as: “You are caring and kind” or a gibberish sentence such as “briggy tublish tuchty”. The participants were asked to categorize the sentence they heard either as meaningless, meaningful, or meaningful and pleasant, and press the corresponding number 1, 2. or 3 on the numerical keypad.

**Video (VDO):** In this sensory modality, ambiguous images were displayed sequentially (see example as illustrated in Figure 1), while the song “Imagine” by John Lennon was played in the background. Like meditation, this was a passive task that required no active response from the participants.

The experiments were conducted inside a Faraday chamber, while the participants were sitting comfortably on a chair, in front of a computer with a keyboard. To minimize artifacts while answering the experimental questions via the space bar and the numerical keypad, participants were asked to attenuate eye, head, and body movements. Three (3) checks were conducted by the experimenters as follows:

- An impedance check prior to the beginning of the experiment.
- A second impedance check after the first two modalities, MED and WORDS (block 1).
- A third and final impedance check after modalities IMG and MM (block 2).
- The experiment concluded with the modalities SENT and VDO (block 3).

We measured twenty (20) healthy participants, eleven (11) males and nine (9) females, between twenty-three (23) and sixty-four (64) years of age. Amongst the participants were eleven (11) meditators varying in expertise and nine (9) non-meditators. The present work excludes the analysis of the significance of meditation experience among the participants; for meditation-related results, see [36]. Here we group meditation and relaxation in the modality MED as described above.

### 2.2. Preprocessing

Data were collected at a sampling frequency of 1000 Hz, using the 128-channel EEG array. The vector of 128 data points at a given time was converted into a 12 × 12 square matrix, corresponding to cortical areas. In order to fill in the open positions in the matrix, some of the data channels were duplicated. Two locations on the prefrontal cortex, A(12,3) and A(12,10), were used as reference positions and left open; see [36] for experimental details.

We used standard preprocessing techniques, including a notch filter at a frequency of 50 Hz, and a detrending filter to reduce movement artefacts [34]. These approaches produced data over the frequency band of 2 Hz to 48 Hz, which served as a starting point for future detailed analysis.

Finally, we mention that a 22 ms adjustment was introduced to align the EEG recordings with the time instances of the recorded events and actions, e.g., pressing a key by the participants. The 22 ms adjustment was required in part due to the delay in the anti-aliasing filter (8 ms), and also due to the screen refreshing rate (14 ms).

### 2.3. Hilbert Analysis

In this section, we briefly summarize the signal-processing methodology using the Hilbert transform, which allows studying the characteristics of rapidly changing signals.

The Hilbert transform methodology has the advantage that it allows for targeting significantly nonstationary signals, with possible nonlinear characteristics in the time domain. Brain signals exhibit strongly nonstationary and nonlinear properties, and Hilbert transform-based methods have been successfully applied to analyze spatio-temporal brain dynamics in the past decades [40]. The Hilbert transform significantly relies on band-passed filtering of the measured data, and the proper design of the filters is an essential component of the approach. Clearly, Hilbert analysis ought to be complemented with additional approaches and information-theoretic measures, based on Fourier analysis and spectral densities, e.g., [36].

By applying the Hilbert transform, each EEG electrode signal s(t) is transformed to **S**(t) as follows:

**S**(t) = s(t) + i s(t)* (1)

(2) $$S\left(t\right)=AA\left(t\right)e^{i\;AP\left(t\right)}$$

where s(t)* = $\frac{1}{\pi }$ p.v. $\int_{-\infty }^{+\infty }\frac{s\left(t’\right)}{\left(t-t’\right)}dt’$ and where p.v. is the Cauchy Principal value.

Here, **AA**(t), **AP**(t), and **IF**(t) stands for analytic amplitude, analytic phase, and instantaneous frequency, respectively. They are defined as:

(3) $$AA\left(t\right)=\sqrt{{s\left(t\right)}^{2}+{s\left(t\right)*}^{2}};\;AP\left(t\right)=\mathrm{atan}\left(\frac{s\left(t\right)*}{s\left(t\right)}\right)$$

(4) $$IF\left(t\right)=\left(\frac{1}{2\pi }\right)\left(\frac{∆AP\left(t\right)}{∆t}\right)=\left(\frac{1}{2\pi }\right)\left(\frac{AP\left(t\right)-AP(t-∆t}{∆t}\right)$$

**Figure 2:**
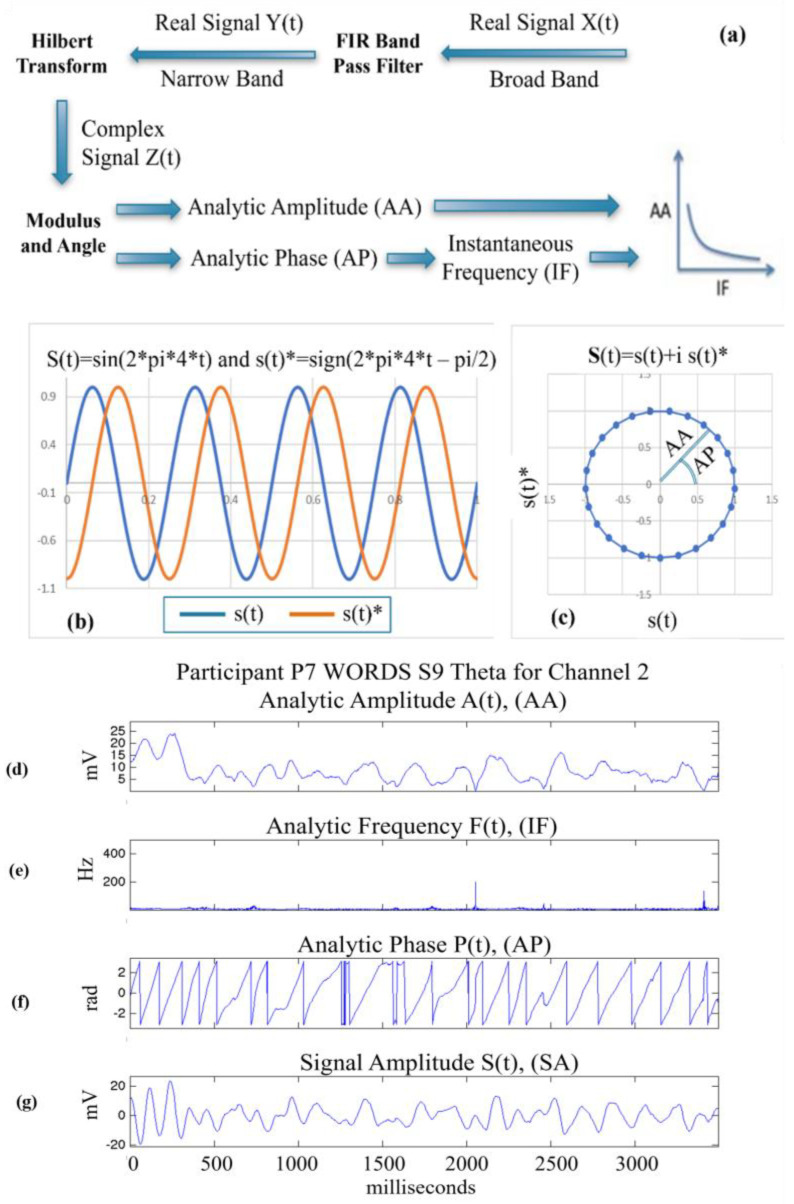
(**a**) Schematics of the Hilbert transform-based methodology when a narrow frequency band is applied to the EEG signal (X_t_), producing the filtered signal Y_t_, followed by a Hilbert transformation. This leads to signal Z_t_, which is complex valued. Considering polar coordinates, the signal is described by its amplitude and phase. The modulus of Z_t_ gives the analytic amplitude (AA), while the angle produces the analytic phase (AP). (**b**) Resulting signals after Hilbert transform is applied to a sinus time series, showing the real and imaginary parts of the complex signal. (**c**) Analytic amplitude (AA) and phase (AP) derived from the resulting signals after Hilbert transform is applied. Examples of the different indices that were computed after Hilbert transforming the signal amplitude for EEG Channel 2: (**d**) **AA**(t), (**e**) **IF**(t), (**f**) **AP**(t), (**g**) **SA**(t).

Figure 2 illustrates the applied Hilbert transform-based signal-processing algorithm [40,45]. As an example, let us define s(t) = sin(ωt), then s(t)*$=sin\left(\omega t-\frac{\pi }{2}\right)\;\forall$ ω < 0 and $sin\left(\omega t-\frac{\pi }{2}\right)$ ∀ ω > 0, and we can visualize **AA**(t) and **AP**(t) as shown in Figure 2b,c. We used the MATLAB “Hilbert” function to compute the imaginary part s(t)* from the real valued signal s(t). In Figure 2d–g we show an example for one of the 144 channels, channel 2, measured via the EEG net.

### 2.4. Computation of the Pragmatic Information Index

Pragmatic information (PI) was introduced originally to describe collective oscillations in laser systems [38]. It is a fundamental extension of the Shannon information, and it has been applied to brain dynamics [39]. Freeman observed that pragmatic information is applicable to describe the sequences of brain amplitude modulation patterns, as order parameters [45]. Pragmatic information, as applied to brain oscillatory patterns, can serve as a biomarker of intentionality and the creation of meaning, and it can be interpreted in terms of many-body field dynamics [41]. In practical terms, the pragmatic information index is given as the ratio of the dissipation of free energy and the rate of change in the indicated order parameter [45]. Let H_e_(t) denote the pragmatic information index, which is expressed as follows:

(5) $$H_{e}\left(t\right)=\langle {AA}^{2}\left(t\right)\rangle /D_{e}\left(t\right)$$

Here, D_e_(t) is the distance between consecutive **AA**(t) patterns, while 〈 〉 is the ensemble average across space. Instead of using the distance between **AA**(t) patterns to derive H_e_(t), we can use **AP**(t) to arrive at an alternative version of the pragmatic information index.

In the case of the EEG experiments, the **AA**(t) vector has 128 components, and the distance between consecutive patterns can be simply calculated using the Euclidean measure as follows:

D_e_(t) = |**AA**^2^(t) − **AA**^2^(t − 1)|**_2_** (6)

In an alternative definition of D_e_(t), the distance between consecutive analytic phase patterns **AP**(t) can be employed, as it is described in Equations (7)–(11). Here, the time index runs through t = 1, 2, …, t*, …, T, while the index for the spatial location of the electrodes will be i = 1, 2, …, 128:

(7) $${∆\;AP(t*)}_{i}={AP\left(t*\right)}_{i}-{AP\left(t*\right)}_{i-1}$$

∀i=2,Nch,for t=t*

(8) $$∆\;AP(t*)={\left({∆\;AP(t*)}_{1},\;{∆\;AP(t*)}_{2},\ldots ∆\;AP(t*\right)}_{Nch}$$

(9) $$D_{e}\left(t*\right)={\left|∆\;AP(t*)\right|}_{2}\forall i=2,Nch$$

(10) $$D_{e}\left(t*\right)=\sqrt{\sum_{i=2}^{Nch}{\left[{∆\;AP(t*)}_{i}\right]}^{2}}$$

(11) $$D_{e}\left(t\right)=\left(D_{e}\left(1\right),D_{e}\left(2\right),\ldots .,D_{e}\left(t*\right),\ldots D_{e}\left(T\right)\right)\forall t=1,T$$

where *i* is a particular channel, t* is a particular point in time, *Nch* is the total number of channels (electrodes), and T is the time length of the signal or last temporal point.

The concept of pragmatic information has been applied to general complex systems [46]. Pragmatic information has a unique potential to characterize meaning and provide a measure to describe brain dynamics [47]. This is especially remarkable as the very foundation of pragmatic information goes back to the work by von Weizsäcker, making a distinction between the Shannonian syntactic information, and the general semantic and pragmatic information [48,49].

**Figure 3:**
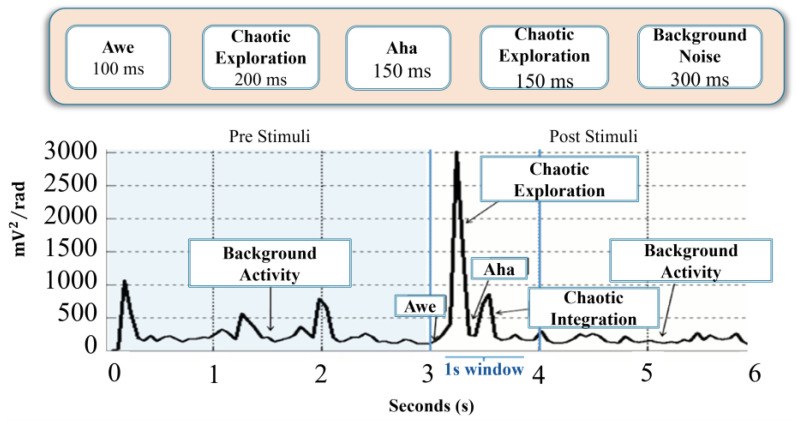
Illustration on the cycle of creation of knowledge and meaning. A visual stimulus is presented to the animal at time instant 3 s. The stimulus is processed and resolved in the 1 s window following stimulus presentation [37].

Using the pragmatic information index to describe brain dynamics based on measuring EEG signals, we may gain insight into the electrical and metabolic processes underlying the creation of meaning in brains. This view is supported by results obtained by ECoG experiments with rabbits trained by the classical conditioning paradigm [21,23]. Detailed re-evaluation of the experiments conducted in the 1990s showed that the pragmatic information index exhibited distinct peaks following an approximately 1 s post-stimulus period. This is illustrated in Figure 3, previously published in [37], where the stimulus happens at time instant 3 s.

During the 1 s post-stimulus period, the following distinct cognitive processing steps are indicated: Awe, Chaotic Exploration, Aha (also called the Eureka moment of sudden insight), Chaotic Exploration, and returning to Background Activity. These steps are hypothesized as parts of the cycle of the creation of knowledge and meaning (CCKM) [50].

In the next section, we present our findings and analysis of human brain dynamics, which show greater complexity than animal brain dynamics. In humans, meaning creation has several levels, which manifest more complexity than the case of perceiving a single salient stimulus in a time window of 1 s, which has been illustrated based on the rabbit ECoG studies outlined in Figure 3.

## 3. Results

### 3.1. Overview of the Multimodal Experiments

This section provides a comprehensive description of the experimental results obtained with six modalities, using the 128-eletrode EEG array placed over the scalp of the participants. Details of measured EEG signals, their analytic amplitudes and phases, as well as the derived pragmatic information, are given.

**Figure 4:**
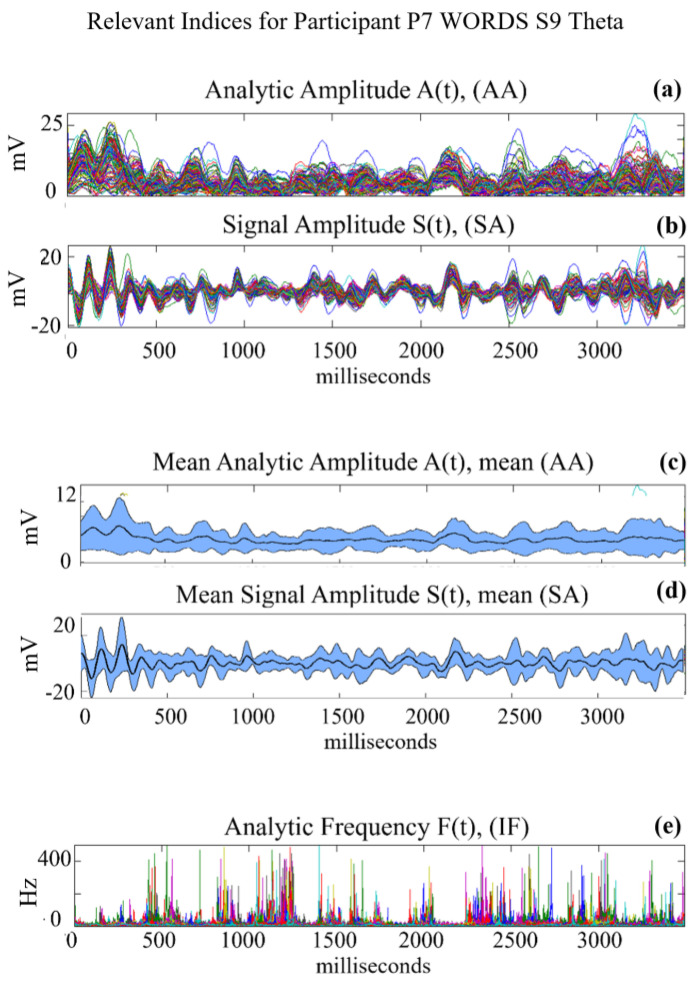
Examples of the different indices that were computed after Hilbert transforming the signal amplitude for each of the 128 electrodes (plotted in different colors in **a**, **b** and **e**): (**a**) analytic amplitude A(t) or **AA**(t), (**b**) signal amplitude S(t) or **SA**(t), (**c**) spatial ensemble averages 〈**AA**(t)〉 with 3-sigma band, (**d**) spatial ensemble averages 〈**SA**(t)〉 with 3-sigma band, (**e**) analytic frequency **IF**(t).

Figure 4 illustrates the analytic amplitude (**AA**(t)), signal amplitude (**SA**(t)), and analytic frequency (**IF**(t)) over a 3.5 s observation period, for frequency band (Theta), for Participant 7. Specifically, Figure 4a,b display the **AA**(t) and **SA**(t) signals for all 128 electrodes, while Figure 4c,d show the average 〈**AA**(t)〉 and 〈**SA**(t)〉 signals calculated across the EEG array, together with the shaded upper and lower limits, respectively. Over the ~2000 ms to 2300 ms time window, we observe significant peaks in **AA**(t) and 〈**AA**(t)〉, indicating that the power of the signal, and therefore its energy, have increased in that time window.

**Figure 5:**
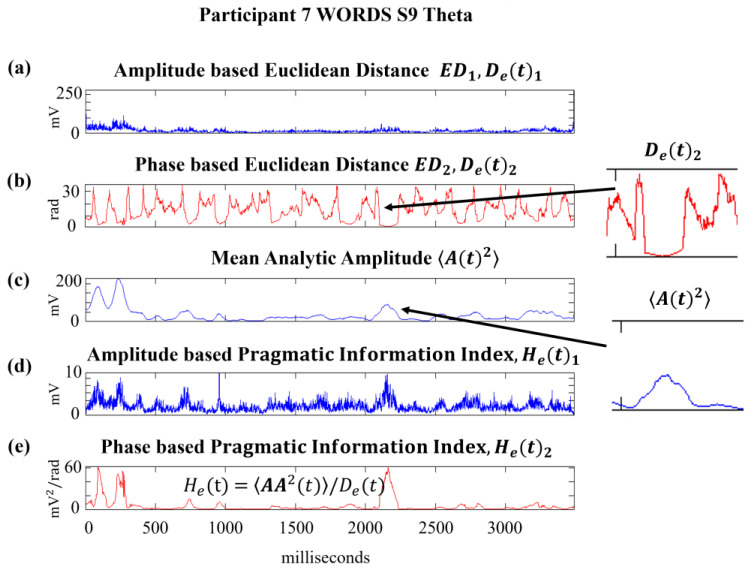
Pragmatic information illustration; (**e**) H_e_(t) is the result of the ratio $\langle A^{2}\left(t\right)\rangle /D_{e}\left(t\right)$ where D_e_(t) and **AA**^2^(t) are shown in plot (**a**,**b**,**c**), respectively. (**d**) displays H_e_(t)_1_ and (**e**) H_e_(t)_2_; these are pragmatic information indices where D_e_(t) is based on amplitude and phase, respectively.

Figure 4e displays the analytic frequency **IF**(t) for the 128 EEG electrodes. The curves show many spikes, which indicate rapid changes in the analytic frequency at various time instances. For our future discussions, it is important to point out that the **IF**(t) values are consistently low during time interval ~2000 ms and 2300 ms, showing that the signals maintain a rather constant phase during this period, leading to only small variations in the analytic frequency. This means that the signals are highly synchronized during this time window, exactly where **AA**(t) had a significant peak, indicating that the power of the signal, and therefore the energy, increased at that time of high synchronization. This observation has important implications for the behavior of the pragmatic information index, as elaborated later in Figure 5.

Figure 5 provides a graphical illustration of the evaluation of the pragmatic information index, H_e_(t), as described above. In order to characterize where and when knowledge and meanings are created to conduct an intentional action, the pragmatic information index, H_e_(t), was evaluated during the 3.5 s response period depicted in Figure 5. We can appreciate the significant changes in pragmatic information, H_e_(t), as a result of computing the ratio $\langle {AA}^{2}\left(t\right)\rangle /D_{e}\left(t\right),$ where D_e_(t) and **AA**^2^(t) show relevant features shaping H_e_(t) dynamics. We display two versions of PI, namely H_e_(t)_1_ and H_e_(t)_2_, where D_e_(t) is based on amplitude and phase, respectively [33].

We produced similar plots for all participants, for all modalities, for all stimuli, in every frequency band of interest, as shown in Table 1. From this first qualitative analysis and based on Freeman’s findings [45], we expect that the most relevant frequency bands with potential impact on the creation of knowledge and meaning are High Gamma (35–48 Hz), and Alpha (8–12 Hz), which is proposed to be acting as a gating band. This is in line with the proposal of Freeman and Kozma and others, suggesting a Gamma–Alpha and Gamma–Theta link [51,52,53,54].

**Figure 6:**
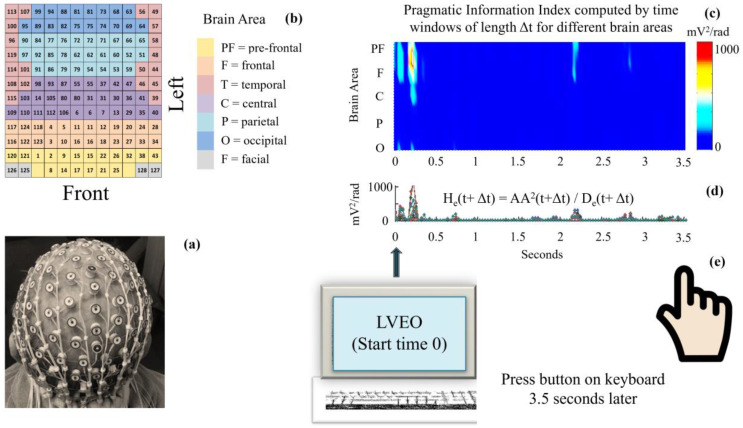
(**a**) Example of the positioning of the EGI EEG array (128 electrodes) on participant’s scalp. (**b**) Brain areas color coded and represented in a matrix. (**c**) Contour plot of the pragmatic information index H_e_(t) during the 3.5 s response time across pre-frontal, frontal, central, and occipital brain areas, as displayed on subplot (**b**); see also [37]; (**d**) shows the H_e_(t) signals for the same brain areas and time windows Δt. (**e**) Both graphs (**c**,**d**) display results for participant P7, stimulus S9, in modality WORDS, for the Theta band, where the stimuli presentation (LVEO) coincides with start time 0, and the pressing of the button to provide an answer takes place at the end of the processing of the stimuli, which coincides with time 3.5 s.

Figure 6 illustrates the EEG in subplots (a)–(b), and an example of the distribution of the pragmatic information index across various brain areas, during a 3.5 s experimental period. The PI plot corresponds to participant 7 (P7), in the modality WORDS, on stimulus 9 (S9), in the Theta band. This example illustrates that the creation of knowledge and meaning is manifested in different combinations of brain areas and frequency bands. These computations for H_e_(t) use a specific implementation of the PI evaluation, where we have performed the evaluation in time windows of length Δt as H_e_(t − Δt).

### 3.2. Evaluation of Pragmatic Information Variables and Parameters

Next, we introduce a set of variables to characterize the dynamics of H_e_(t) during the time period necessary to process a particular stimulus [37], which are given as follows:

- NPS: the total number of peaks per unit time (s);
- TBP: the time spent between peaks, describing the quiet periods;
- TOP: the time describing the duration of peaks, measuring intensive periods;
- QPT: the total quiet processing time;
- IPT: the total intensive processing time.

The number of peaks will be described by the following aggregate quantity $\gamma_{b,e\;}^{p,m,s}$, where the indices stand for stimulus (*s*), participant (*p*), modality (*m*), frequency band (*b*)*,* and electrode or channel (*e*), where p, m, b, e, and s can take values as shown in Table 2. Table 2 shows that there are different numbers of stimuli in different experiments, as specified by ${Ns}^{m}$ in Table 3.

Integrating over all channels, *Nch* = 128, we obtain the total number of peaks in an experiment with the following parameters: stimulus (*s*), participant (*p*), modality (*m*), and frequency band (*b*):

(12) $${\tilde{\gamma }}_{b}^{p,m,s}=\sum_{e=1}^{Nch=128}\gamma_{b,e\;}^{p,m,s}$$

From ${\tilde{\gamma }}_{b}^{p,m,s}$, NPS can be derived as the number of peaks per second, for given stimulus (*s*), participant (*p*), modality (*m*), and frequency band (*b*), based on the total duration:

(13) $${\dot{\gamma }}_{b}^{p,m,s}=\frac{{\tilde{\gamma }}_{b}^{p,m,s}}{t_{s}^{p,m}}$$

Considering all the combinations of frequency bands (b) and modalities (m), there are 36 possibilities; we can evaluate the mean values over all stimuli, ${\bar{\dot{\gamma }}}_{b}^{p,m}$ as follows:

(14) $${\bar{\dot{\gamma }}}_{b}^{p,m}=\sum_{s=1}^{{Ns}^{m}}{\dot{\gamma }}_{b}^{p,m,s}/{Ns}^{m}$$

From ${\bar{\dot{\gamma }}}_{b}^{p,m}$ we can derive (a) the number of peaks/second for participant *p*, in modality *m* for all frequency bands as ${\bar{\dot{\gamma }}}^{p,m}$, and (b) the number of peaks/second for participant *p*, in frequency band *b* for all modalities as, ${\bar{\dot{\gamma }}}_{b}^{p}$ as follows:

(15) $${\bar{\dot{\gamma }}}^{p,m}=\sum_{b=1}^{6}{\bar{\dot{\gamma }}}_{b}^{p,m}/6$$

(16) $${\bar{\dot{\gamma }}}_{b}^{p}=\sum_{m=1}^{6}{\bar{\dot{\gamma }}}_{b}^{p,m}/6$$

Similarly, we compute intervals of confidence based on the standard deviation, ${S\dot{\gamma }}^{p,m}$ for the values of ${\bar{\dot{\gamma }}}^{p,m}$, as well as intervals of confidence based on the standard deviation, ${\;S\dot{\gamma }}_{b}^{p}$ for the values of ${\bar{\dot{\gamma }}}_{b}^{p}$, as follows:

(17) $${\bar{\dot{\gamma }}}^{p,m}\pm \;t_{\propto =0.05,6\;}*{S\dot{\gamma }}^{p,m}/\sqrt{6}$$

(18) $${\bar{\dot{\gamma }}}_{b}^{p}\pm \;t_{\propto =0.05,6\;}*{S\dot{\gamma }}_{b}^{p}/\sqrt{6}$$

Similar formulas can be derived over brain areas, when the number of electrodes over the specific area are used. For example, the following expressions are valid for the prefrontal region PF:

(19) $${\tilde{\gamma }}_{b,Fp}^{p,m,s}=\gamma_{b,1}^{p,m,s}+\gamma_{b,2}^{p,m,s}+\gamma_{b,8}^{p,m,s}+\ldots \;\;\forall \;e\;\in Fp$$

Considering all areas, we write:

(20) $${\tilde{\gamma }}_{b,B_{a}}^{p,m,s}=\gamma_{b,n1}^{p,m,s}+\gamma_{b,n2}^{p,m,s}+\ldots +\gamma_{b,nn}^{p,m,s}$$

$$\forall e=n1,n2,\ldots nn\in B_{a}\;where$$

$$B_{a}=Fp,F,T,C,P,O,Facial$$

Following the approach described above, formulas can be derived for the other statistical quantities described earlier, such as TBP, TOP, QPT, and IPT. When needed, the corresponding formulas will be provided in the section of analysis.

When characterizing H_e_(t), the following specifications are used:

- A threshold signal value is determined heuristically; a specific threshold is set at 0.1 in these studies.
- Time of peak (TOP) for each significant peak is defined as the duration of the signal continuously above such threshold.
- Time between peaks (TBP) is defined as the duration of the signal continuously below the threshold.
- The selection of a minimum TBP, in this study ≤11 ms, where two consecutive peaks should be taken and joined as one peak.
- Isolated peaks that are too short, here shorter than ≤50 ms, are rejected.

We start by setting a threshold value for the pragmatic information index [29]. Most of the time PI has sub-threshold values, but at some moments it may cross to super-threshold values. Such crossing events are used as markers for the postulated emergence of meaning related to the specific sensory stimuli in the context of the participants’ physical and cognitive state, and their intentions and desired actions. These are precisely the moments that may be considered to consume the most energy. Details of the energy consumption during the cognitive cycle require further studies and are outside the scope of this work; see, e.g., [29].

**Figure 7:**
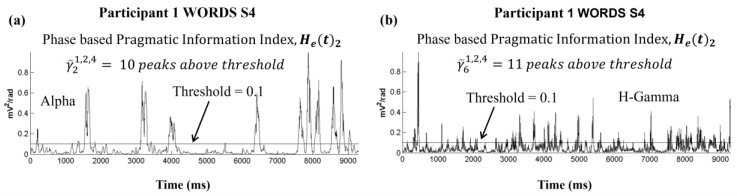
Pragmatic information index H_e_(t) for (**a**) in the Alpha and (**b**) the H-Gamma frequency bands; illustrating peaks above threshold 0.1, with selection rules considering peak duration and time between peaks.

Figure 7 shows an example with a PI threshold set at 0.1, for frequency band (a) Alpha (b = 2) and (b) High Gamma (b = 6); these examples correspond to the fourth stimulus (s = 4) in the WORDS modality (m = 2). We observe by visual inspection 10 peaks in the case of the Alpha band, and 11 peaks for the H-Gamma band. Using the compact index notation, this is expressed as ${\tilde{\gamma }}_{b=2}^{p=1,m=2,s=4}=10$ and ${\tilde{\gamma }}_{b=6}^{p=1,m=2,s=4}$ = 11. Several peaks may be merged when counting their numbers if they are very close to each other, or rejected when they are individually very short. Details of the practical implementations are given in the next section.

### 3.3. Results Based on NPS

**Figure 8:**
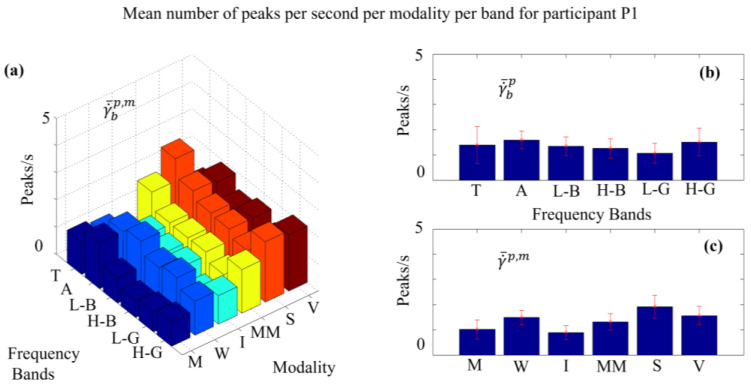
(**a**) Comprehensive illustration of **NPS** ${\bar{\dot{\gamma }}}_{b}^{p,m}$ across six modalities and six frequency bands, in the case of participant 1. The modalities are in the same order as introduced at the beginning, e.g., Meditation (M) first in dark blue and MathMind (MM) fourth in yellow. (**b**) Mean peaks/second ${\bar{\dot{\gamma }}}_{b}^{p}$, across modalities, for the six frequency bands; (**c**) mean peaks/second ${\bar{\dot{\gamma }}}^{p,m}$ across frequencies, for the six modalities.

An important statistical index derived from the pragmatic information index is the number of peaks per second (NPS). In Figure 8a we display the mean NPS per modality and frequency band for participant P1. We observe that the mean NPS, ${\bar{\dot{\gamma }}}_{b}^{1,m}$, varies across modalities and frequency bands. For example, the modality SENT shows more peaks/second than the other modalities. In the case of MED modality, there is a general tendency of having higher NPS over the Theta and Alpha bands, as compared to the rest of the bands. Visual inspections indicate remarkable differences between various participants, and also commonalities between participants over modalities and frequency bands. Detailed statistical analysis can reveal the significance of such observations, as described next.

In order to illustrate the difference in peaks/second for each modality, ${\bar{\dot{\gamma }}}^{p,m}$, we produce a bar graph in Figure 8c, after summing up over all frequencies; see Equation (17) for details. Figure 8b displays the mean peaks/second, ${\bar{\dot{\gamma }}}_{b}^{p}$, for each frequency band, after summing up over all modalities, according to Equation (18).

**Figure 9:**
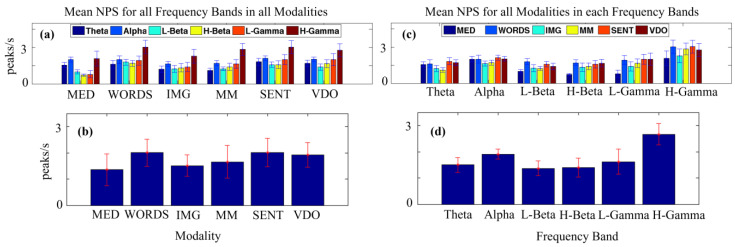
Results of NPS evaluations: (**a**) mean NPS for various frequencies, clustered according to the six modalities; (**c**) mean NPS for various modalities, clustered according to the frequencies. Mean NPS with error bars across participants for (**b**) modalities and (**d**) frequency bands.

The results regarding the NPS values combined over all participants are shown in Figure 9, which shows the differences with respect to modalities, as well as frequencies. The observations are summarized as follows:

1. In Figure 9c, the Alpha and H-Gamma frequency bands show the highest mean NPS values for all H-Gamma and most Alpha values across participants, when we compute the mean and confidence intervals for each modality in each frequency band. This points to the Alpha and H-Gamma linkage established in other studies [6,55].
2. Except for Theta and Alpha frequency bands, modality MED shows the lowest mean NPS values. This should be expected since the Theta and particularly the Alpha frequency bands have been shown to be the dominant frequencies in meditative states in previous studies [36]. However, this needs more investigation.

At the next level of granulation, we combine either all frequency bands together in each modality, Figure 9b, or all modalities in each frequency band, Figure 9d. Main observations are as follows:

1. Modalities MED and IMG show lower NPS values when compared to all other modalities; Figure 9b.
2. H-Gamma and Alpha frequency bands show the highest NPS values, see Figure 9d.
3. The NPS measure, it seems to us, is a good candidate for the estimation of intensive processing periods, and likely will be suitable to differentiate between high and low energy consumption modalities.

### 3.4. Results Obtained with TBP and TOP

**Figure 10:**
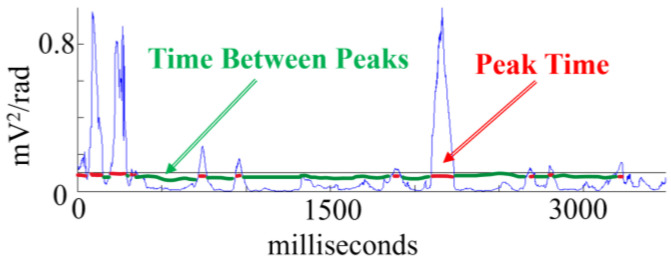
Illustration of the quantities time between peaks (TBP) in green, and time or duration of a peak (TOP), in red. The blue line shows the computed PI index H_e_(t)_2_.

Another set of relevant variables is described by TBP, which is the time between peaks, and it is a measure of quiet processing. We also introduce results for TOP, which is the time of peaks, and it corresponds to time periods with intensive processing; see Figure 10 for illustrations.

**Figure 11:**
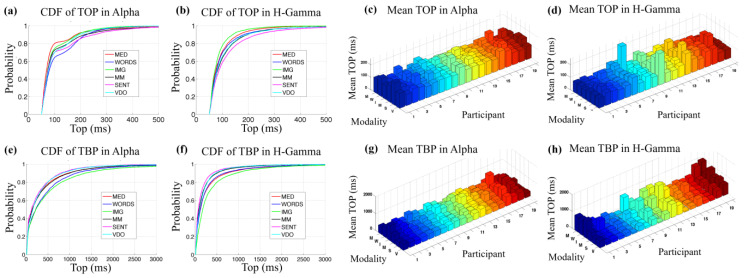
Upper row shows CDF plots for TOP values in (**a**) the Alpha and (**b**) the H-Gamma frequency band for all participants in each modality; (**c**) shows 3D bar graphs of mean TOP values for each participant (*x*-axis) and each modality (*y*-axis) for the Alpha frequency band and (**d**) for the H-Gamma frequency band. Lower row shows CDF plots for TBP values in (**e**) the Alpha and (**f**) the H-Gamma frequency band for all participants in each modality; (**g**) shows 3D bar graphs of mean TBP values for each participant (*x*-axis) and each modality (*y*-axis) for the Alpha frequency band and (**h**) for the H-Gamma frequency band. The different colors in (**c**,**d**,**g**,**h**) represent the 20 participants from P1 (dark blue) to P20 (dark red).

TOP and TBP are used to describe the intensive and the quiet processing periods, respectively. At this time, no systematic hypothesis tests were performed regarding TOP and TBP values across modalities. Nonetheless, several observations are useful. For example, when analyzing the cumulative distributions for TOP, as shown in Figure 11, for each modality, in the Alpha (a) and H-Gamma (b) frequency band for all participants, we observe that the TOP values for the modalities of SENT and WORDS seem to behave differently from other modalities, and appear to be greater than the values for the modalities of MED and IMG for the Alpha band. Regarding the H-Gamma frequency band, the values for the modalities of MED and IMG seem to be significantly lower only in relation to the SENT modality, while the modality WORDS behaves more similarly to the modalities of MM and VDO.

The cumulative distribution of the Alpha frequency band (a) may indicate a bimodal or possibly multimodal distribution, which could be due to the fact that some stimuli have longer duration than others for different participants in a particular modality, meaning that some participants may be slower to resolve a task than others. This bimodal or multimodal behavior seems to be absent for most of the modalities in the H-Gamma frequency band. The bimodal or multimodal behavior observed in the Alpha for TOP values is also observed in each frequency band for most modalities, and this could also be associated with the behavior of different brain regions. The study of the behavior of different regions will be the objective of future studies. In general, the mean TOP for each participant shows similar values between the Alpha (c) and the H-Gamma (d) frequency bands, with slightly more variability in the H-Gamma frequency band.

The cumulative distributions for TBP are shown in the lower row (e–h) in Figure 11. Qualitative observations indicate that the distributions of TBP in all modalities for all participants seem to exhibit smaller values for H-Gamma (f) than for Alpha (e), with the exception of the modality MED. In general, several of the mean TBP values are higher for the Alpha (g) than for the H-Gamma (h) frequency band, except for some exceptions like participants 19 and 20, for example. Note that the H-Gamma frequency band displays more variability.

**Figure 12:**
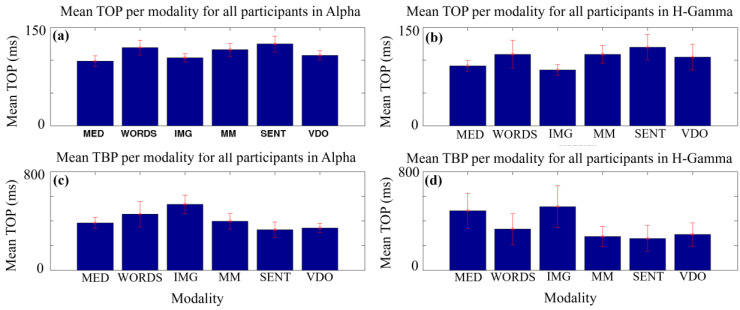
Mean TOP (**a**,**b**) and mean TBP (**c**,**d**) values with corresponding error bars for all participants and each modality in the Alpha frequency band (**a**,**c**) and the H-Gamma frequency band (**b**,**d**).

Finally, in Figure 12 we summarize the above analysis by showing the mean TOP (a,b) and TBP (c,d) for each modality, for all participants, in the Alpha (a,c) and H-Gamma (b,d) frequency bands. We can observe that, for TOP, the values are smaller for the modalities of MED and IMG, while SENT and WORDS display the largest values for both the Alpha and the H-Gamma frequency bands. For TBP, we find a more diverse range of values across modalities. IMG displays large values for both frequency bands. However, MED, and SENT and VDO, have the lowest values for the Alpha frequency band, and it is MM and SENT that display the smallest values for the H-Gamma frequency band. Again, these qualitative visual observations must be evaluated using systematic hypothesis tests in the future.

**Figure 13:**
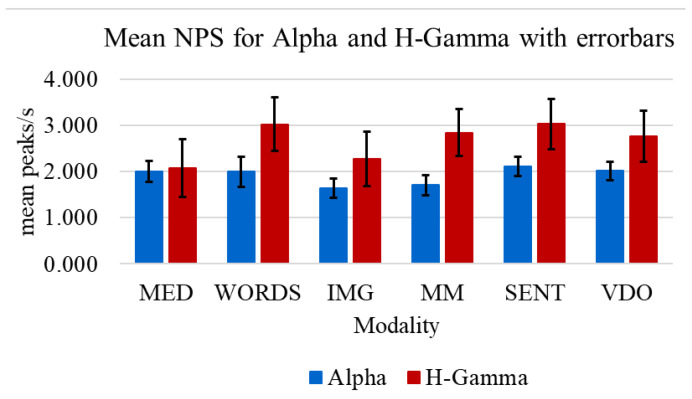
Mean NPS with error bars for all participants in each modality for the Alpha and the H-Gamma frequency bands.

Regarding the mean NPS, some interesting observations can be made when contrasting the Alpha and H-Gamma frequency bands, as shown in Figure 13 and Table 4. Table 4 uses data from [37], but the modalities have been regrouped, to emphasize the significance of the observations on low NPS values in IMG, MEG, and MM modalities.

In the Alpha frequency band, we can clearly identify the two (2) minimum mean NPS values associated with the modalities IMG and MM, while for the H-Gamma frequency band, the two (2) minimum values are identified for the modalities MED and IMG. The common minimum of the two frequency bands is found in the modality IMG. In the IMG task, the participants are less in control of the process of knowledge creation, until the moment of sudden insight, the “aha” moment, when they identify the meaning of the earlier hidden image. This, it seems to us, would require less total intensive processing time, which is a combination of the mean TOP and the mean NPS, as previously explained in Figure 10, if we add all the TOP values (red) of each peak above the threshold.

We expected that the TOP values for the modality MED would be smaller than for the rest of the modalities, since meditation practices are known to reduce information processing in the brain, and therefore the creation of knowledge and meaning would also be reduced, more likely leading to less NPS and shorter TOP. What is interesting, however, is that we observe a similar behavior for IMG, which suggests that between the sudden discoveries of meaningful images, there is little processing of semantic information, which coincides with longer periods of less intensive processing, as seen in TBP values for both the Alpha and H-Gamma frequency bands.

Based on the parameters (mean and standard deviation) used to compute the confidence intervals displayed in Table 4, we performed a set of unequal variance *t*-tests of hypothesis, where H0: μ1 = μ2 was tested for means between all modalities in the Alpha and H-Gamma bands. The results are displayed in Table 5. We also tested for means between the Alpha and H-Gamma bands for each modality. It is important to note that we accept H0 for *p*-values greater than or equal 0.05, while we reject it for values smaller than 0.05. An accept is labeled as {0} and a reject as {1}, and a {0*} indicates a just accepted with a value marginally greater than 0.05, like, for example, 0.06.

From Table 5, it is clear that the modality MED shows a significant number of rejects (1) and just accept (0*), particularly for the H-Gamma band, when compared with the rest of the modalities. For example, we observe that the NPS means for the modality WORDS show the same mean, statistically speaking, when compared to the rest of the modalities in both bands, except for modality MED in the H-Gamma band.

When taking the results of the Alpha and H-Gamma bands together, we reject H0 when comparing the mean NPS values of modality MED with the rest of the modalities, except for the modality VDO where H0 is accepted (0) and MM is just accepted (0*) when compared with MED. In the case of modality WORDS, H0 is accepted (0) for all but modality MED. For modality MM, H0 is accepted (0) when compared with most modalities and only rejected (1) for modalities SENT and VDO in the Alpha band. Additionally, H0 is rejected when contrasting the modalities of IMG with SENT and VDO for the Alpha band.

In H-Gamma, all other combinations of means hypothesis testing return a value of 0, accepting that means between modalities are equal, except when contrasted with modality MED.

When we test H0: μ1 = μ2 for a comparison between the Alpha and H-Gamma bands, we accept H0 for *p*-values greater than or equal 0.05, otherwise H0 is rejected. It is important to note that we rejected H0 for mean values of NPS for all modalities except for modality MED.

In order to have an idea of the percentage of time that the brain is engaged in intensive processing, as well as the percentage of time that it is operating in quiet processing mode, we computed a pair of variables, as follows.

Let us define (1) $T_{b}^{p,m,s}$ as the duration of stimuli s, for modality m, for participant p, in frequency band b; (2) ${IPT}_{b}^{p,m,s}$ as the total intensive processing time in stimuli s, for modality m, for participant p, in frequency band b; and (3) ${NP}_{s}$ as the number of peaks per stimuli s, for modality m, for participant p, for a particular frequency band b*. It follows that:

(21) $${IPT}_{b*}^{p,m,s}=\sum_{k=1}^{{NP}_{s}}{TOP}_{b*,k}^{p,m,s}$$

(22) $${PIPT}_{b*}^{p,m,s}=\frac{{IPT}_{b*}^{p,m,s}}{T_{b*}^{p,m,s}}$$

where ${PIPT}_{b*}^{p,m,s}$ is the percentage of intensive processing time in one stimulus, for modality m, participant p in a particular frequency band b*.

We then compute a vector ${PIPT}_{b*}^{m}$ where each particular ${PIPT}_{b*}^{p,m,s}$ becomes an element of this vector, for modality m, in a particular frequency band b*, and we use the values in this vector to compute and plot the cumulative probability distributions of ${PIPT}_{b*}^{m}$ for each modality m.

Similarly, we compute the percentage of quiet processing time for each modality m, for a particular frequency band b*, as vector ${PQPT}_{b*}^{m}$ to produce further statistical analysis.

**Figure 14:**
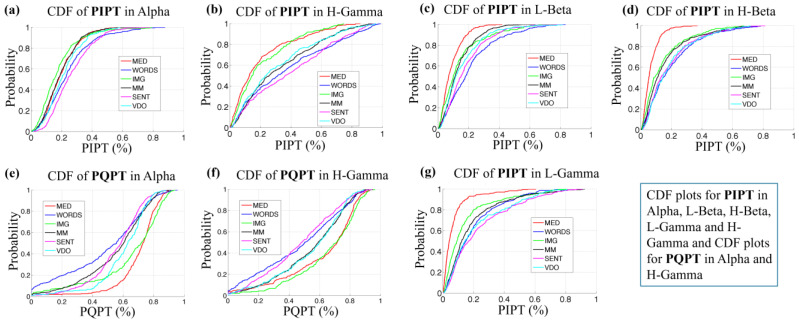
CDF for PIPT, considering all modalities, in Alpha (**a**), H-Gamma (**b**), L-Gamma (**e**), L-Beta (**f**) and H-Beta (**g**), and for PQPT in Alpha (**c**) and H-Gamma (**d**) frequency bands.

In Figure 14a,b,e,f we show the empirical cumulative probability distributions for both ${PIPT}_{b*}^{m}$ and ${PQPT}_{b*}^{m}$ for b* = [Alpha, H-Gamma], for each modality m. As expected, we can observe almost a mirror image of the cumulative probability distributions ${PIPT}_{b*}^{m}$ (a, b) and ${PQPT}_{b*}^{m}$ (e, f) for all modalities in both bands.

We also observe that the ${PIPT}_{b*}^{m}$ values for modality MED and IMG are smaller than the values for the modality of VDO and this modality in turn shows values smaller than modalities SENT and WORDS for both bands. The modality MED behaves like MM in the Alpha frequency band (a), while the modality of MED follows a closer behavior to the modality of IMG in the H-Gamma (b). It is also clear that the modalities are more clustered in Alpha than H-Gamma, making H-Gamma a better band to discriminate between modalities.

We also observe in Figure 14c,d,g for the ${PIPT}_{b*}^{m}$ values for b* = [L-Beta, H-Beta, L-Gamma] how clearly the cumulative probability distribution shows the difference between modalities, where the values for MED < IMG < MM < WORDS < VDO < SENT are smaller, in that order, with some small variability for L-Beta. These frequency bands meet the expectation that the modality MED would show the lowest processing times, since participants are expected to be in a calmer and quieter information-processing state of mind.

**Figure 15:**
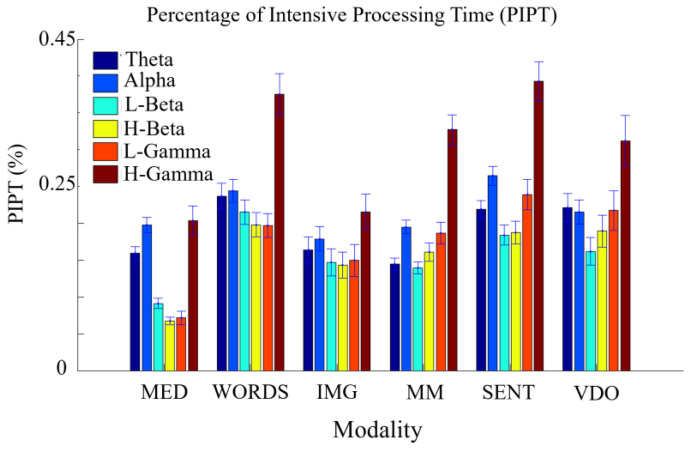
PIPT mean values with error bars, for all modalities in each frequency band.

In Figure 15, we show the mean and errors for the values of the ${PIPT}_{b*}^{m}$, for all modalities and frequency bands, and identify an order from the least to the most intensive processing time modalities, as follows: MED, {MM, IMG}, VDO, {SENT, WORDS}.

Here we identify the smallest and largest PIPT values per modality and per band as displayed in Table 6.

From Table 6, we observe how MED displays the lowest values of PIPT for most bands, apart from Alpha. This could be because in MED, Alpha is the most dominant frequency and less entropic band for most participants, as shown in [36]. Note that modalities MED, IMG, and MM show the smallest PIPT values for all frequency bands, in contrast with the modalities of WORDS, SENT, and VDO, presumably indicating that the first group is less time and energy consuming out of all modalities.

**Figure 16:**
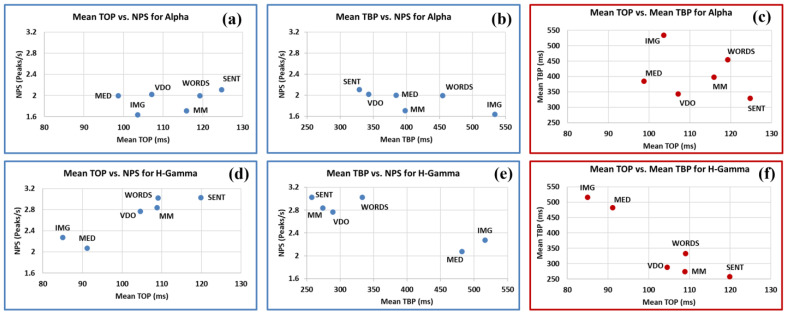
The relationships between mean TOP, NPS, and TBP: (**a**) mean TOP vs. NPS values for Alpha and H-Gamma (**d**), TBP vs. NPS values for Alpha (**b**) and H-Gamma (**e**) and TOP vs. TBP (in red) for Alpha (**c**) and H-Gamma (**f**).

Finally, we present in Figure 16 the big picture about TBP, TOP, and NPS and their relationship to one another for all participants, for all modalities, in the Alpha and H-Gamma frequency bands. As important as they might be in giving us a fuller picture of brain dynamics, we leave the study of other frequency bands for future research since (a) we have still to gain more insight into the Alpha–H-Gamma linkage and (b) because it would make this paper exceed the acceptable length.

As we can appreciate in Figure 16a,d, generally speaking, the mean values of TOP drop in H-Gamma when compared to those in Alpha for all modalities except for VDO, while the values for NPS increase significantly for all modalities (except for MED showing a slight increase) in H-Gamma when compared to the Alpha frequency band.

On the other hand, when we observe the mean values of TBP (b, e) they show a diverse behavior between modalities. Modality MED shows a significant increase and clusters together with modality IMG for H-Gamma when compared to the Alpha frequency band. However, modalities SENT, MM, VDO, and WORDS show a large decrease in H-Gamma when compared to the Alpha frequency band.

In Figure 16c,f we note an inverse relationship between the mean TOP and the mean TBP values, with a larger variability in Alpha, where we also see three (3) clusters of modalities as follows: {MED, IMG}, {MM, WORDS, VDO} and {SENT}.

Based on the above, we constructed Table 7 in order to have a concise picture of the behavior of the different modalities in the Alpha and H-Gamma frequency bands in terms of mean TOP (MTOP), mean TBP (MTBP), and NPS. This allows us to qualitatively characterize these variables, from the smallest to the largest values, that influence the percentage of intensive and quite processing, PIPT, and PQPT respectively.

Taken together, the Alpha and H-Gamma frequency bands show MTOP values displaying an increasing order from the modalities of MED and IMG (1st), VDO (2nd), MM (3rd), WORDS (4th), and SENT (5th). However, when we look at the MTBP values, the different modalities behave in a different manner when contrasting Alpha and H-Gamma, as follows: (a) SENT (1st), VDO (2nd), MED (3rd), MM (4th), WORDS (5th), and IMG (6th) for Alpha, and (b) SENT (1st), MM (2nd), VDO (3rd), WORDS (4th), MED (5th), and IMG (6th) for H-Gamma.

Note that modalities MM and MED show the larger changes in mean TBP values when contrasting their values for the Alpha and H-Gamma frequency bands.

For the NPS, the order is as follows: (a) IMG, MM, WORDS, MED, VDO, and SENT for the Alpha frequency band; and (b) MED, IMG, VDO, MM, SENT, and WORDS for the H-Gamma frequency band. Note that both frequency bands show very distinct orders.

We observe a general tendency of inverse correlation between the variables MTBP and NPS, except for some exceptions in the H-Gamma band, particularly for modality WORDS.

These variables describe very particular aspects of brain dynamics related to velocity of processing per stimulus and the mean intensive processing time per stimulus that can be derived from ${IPT}_{b*}^{p,m,s}$ in Equation (21), which also can be estimated as the mean TOP multiplied by the mean NP per stimulus for all participants, for all modalities, for each band. This analysis could be extended to differentiate between experienced meditators and participants with little or no meditation experience. This issue is not addressed in the present study.

Note that this analysis is based on mean values. In the future, this study should be complemented following the results obtained in Table 5, and further statistical analysis, where a test is performed for H0: μ1 < μ2 or H0: μ1 > μ2, for NPS and other variables.

## 4. Discussion

Based on a comprehensive evaluation of EEG measurements conducted with the participation of 20 volunteers, we explored the brain response to a range of sensory and task modalities, following the pioneering work by Freeman on intentional neurodynamics and the creation of meaning [23,30]. The results presented in this work help better understand the energy aspects of brain dynamics that may lead to a healthy and meaningful life. Several main conclusions are as follows:

- Several statistical indices were introduced based on pragmatic information (PI) to characterize brain dynamics over the Theta, Alpha, Low Beta, High Beta, Low Gamma, and High Gamma bands. We defined the following variables over each band and each modality: number of PI peaks per second (NPS), time between peaks (TBP), time of peak (TOP), quiet processing time (QPT), and intensive processing time (IPT). We conducted a thorough statistical analysis of these variables and found important differences and similarities between modalities and bands.
- The analysis showed that H-Gamma and Alpha frequency bands demonstrate high NPS across the pool of 20 participants. Brain dynamics variables, in both the Alpha and H-Gamma frequency bands, served as classifiers for the different behaviors observed between modalities. This result provides a novel quantitative support to the previously established relationship between Alpha and H-Gamma bands using alternative approaches.
- Except for Theta and Alpha frequency bands, the meditation (MED) modality shows the lowest mean NPS values. This observation is in accordance with other studies showing that meditative states significantly rely on processes over the Theta and Alpha bands. This topic requires further detailed investigation and rigorous statistical hypothesis testing, which are beyond our present work.
- A significant, novel aspect of our PI-based statistical analysis is that the derived information-theoretical indices can be considered as promising candidates for the estimation of intensive processing periods in brains, potentially suitable to differentiate between high and low energy consumption modalities. To compare modalities and characterize their behavior, we may use complementary measures such as information and entropy indices.
- Having a robust experimental tool to non-invasively monitor the energy consumption of brain operational modalities will be very useful for the analysis of cognitive processing in healthy brains, with minimal interference in the person’s daily activities. Moreover, deviations from well-established patterns of activities may help to identify and rectify potential pathological conditions.

In recent years, the important role of rhythmic breathing on psychophysiological coherence has been clearly identified [56,57], including the respiratory modulation of cognitive functions in epileptic patients [58]. The delicate balance of various rhythms in the human body and the relationship to general well-being [59] has been documented, including a comprehensive study on heart rhythm synchronization with the Earth’s time-varying magnetic field [60]. The insights gained in our present study using EEG measurements involving multiple cognitive modalities indicate potential applications to address various cognitive neuroscience challenges, including brain studies associated with mental conditions and brain diseases. Intentions and decisions can shape our dietary, exercise, and relaxation habits, with lasting consequences on health in general. Having a clear understanding of the effect of such intentions, decisions, and habits can help in preventing and, perhaps, even correcting, illness and traumatic experiences.

The main focus of the present study is on meditative states and their potential positive influence on general well-being, closely related to our values and meaningful intentional actions. When we value someone or something, this person or thing becomes meaningful to us. In turn, we value what is meaningful to us, like a spiritual experience, the love of our lives, or our general well-being. Spiritual or universal values like Love, Truth, and Unity seem to be very meaningful as abstractions and experiences that we reflect in social relationships and contracts like marriages and constitutions. Together with inner transformations, new insights, and higher meanings, we can refine our intentions and continuously redefine our goals, in order to improve ourselves in many dimensions.

Meanings are veiled to brain dynamics measurements, yet they are very salient and revealed in human experience. This forces us to consider that the creation and valuation of our most precious meanings ought to be understood by first- and third-person perspective science, which means the study of brain dynamics, hand-in-hand with personal reports and anecdotal veridical evidence based on subjective experience. We hope these studies help to improve the conditions for enhancing human potential, well-being, and meaningful behavioral responses, something that could be explored both experientially and scientifically from an early age.

We dedicate this work to the memory and the spirit of our beloved friend and collaborator Walter J. Freeman.

## Figures and Tables

**Table 1:** Relevant frequency ranges and bands studied.

Frequency Range	Frequency Band
4–7 Hz	Theta
8–12 Hz	Alpha
13–17 Hz	Low Beta
18–25 Hz	High Beta
26–34 Hz	Low Gamma
35–48 Hz	High Gamma

**Table 2:** Values for p, m, b, e, and s as used in the computations.

Participants: P1, P2, …, P20	*p_max_ =* 20
Modalities: MED, WORDS, …, VDO	*m*_max_ = 6
Frequency Bands: Theta, …, H-Gamma	*b*_max_ = 6
Electrodes/Channels: 1, 2, 3, …, 128	*e*_max_ = 128
Stimuli per modality: 1, 2, 3, … , Ns^m^	*s_max_ =* Ns^m^

**Table 3:** Number of stimuli per modality, Ns^m^.

Modality	MED	WORDS	IMG	MM	SENT	VDO
Ns^m^	20	20	12	28	20	10

**Table 4:** NPS values averaged over all participants and all modalities; frequency bands Alpha and High Gamma.

Modality	Significance of Band	Alpha Band	H-Gamma Band
Images (IMG)	Alpha, H-Gamma	**1.64 +/− 0.21**	**2.28 +/− 0.59**
Meditation (MED)	H-Gamma	2.00 +/− 0.23	**2.07 +/− 0.62**
Math Mind (MM)	Alpha	**1.71 +/− 0.21**	2.84 +/− 0.51
Words (WORDS)	None	1.99 +/− 0.33	3.02 +/− 0.58
Sentences (SENT)	None	2.11 +/− 0.20	3.03 +/− 0.54
Video (VDO)	None	2.02 +/− 0.20	2.77 +/− 0.55

**Table 5:** Results of the hypothesis tests between modalities. A number in the set {0,1} that corresponds to a value of the set {accept, reject} respectively, as a result of an unequal variance *t*-test of hypothesis, where H0: μ1 = μ2, allowing for a comparison between a pair of modalities for the Alpha (above matrix diagonal, color yellow) and H-Gamma bands (below matrix diagonal, color blue) based on the NPS index. The symbol “*” indicates a value very close to α = 0.05 (0* means accepted, just).

Vs.	MED	MM	WORDS	SENT	IMG	VDO
**MED**	.	0*	0	0	1	0
**MM**	0*	.	0	1	0	1
**WORDS**	1	0	.	0	0	0
**SENT**	1	0	0	.	1	0
**IMG**	0	0	0	0*	.	1
**VDO**	0*	0	0	0	0	.

**Table 6:** The smallest and largest PIPT values for all modalities in each frequency band.

	Theta	Alpha	L-Beta	H-Beta	L-Gamma	H-Gamma
**Smallest**	MM, MED	IMG, MM	MED, MM	MED, IMG	MED, IMG	MED, IMG
**Largest**	WORDS, VDO	SENT, WORDS	WORDS, SENT	WORDS, VDO	SENT, VDO	SENT, WORDS

**Table 7:** Comparing modalities and frequencies for several indices. The index of ordering from the smallest to the largest, based on the mean values of TOP (MTOP), the mean values of TBP (MTBP), and NPS values as derived from Figure 16, per modality for the Alpha (A) and H-Gamma (G) frequency bands.

MOD	MED		WORDS		IMG		MM		SENT		VDO	
**BAND**	**A**	**G**	**A**	**G**	**A**	**G**	**A**	**G**	**A**	**G**	**A**	**G**
**MTOP**	1	2	5	5	2	1	4	4	6	6	3	3
**MTBP**	3	5	5	4	6	6	4	2	1	1	2	3
**NPS**	4	1	3	6	1	2	2	4	6	5	5	3

## Data Availability

The EEG datasets generated for this study may be available upon request, provided that the proper approval is granted by Professor Ian J. Kirk, Head of Ian J. Kirk’s Lab at the Centre for Brain Research at The University of Auckland in New Zealand.
